# Dog ecology and demography in Antananarivo, 2007

**DOI:** 10.1186/1746-6148-5-21

**Published:** 2009-06-01

**Authors:** Maherisoa Ratsitorahina, Jhon H Rasambainarivo, Soloherilala Raharimanana, Hary Rakotonandrasana, Marie-Perle Andriamiarisoa, Fidilalao A Rakalomanana, Vincent Richard

**Affiliations:** 1 Unité d'Epidémiologie, Institut Pasteur de Madagascar, BP 1274, Antananarivo 101, Madagascar; 2 Département de médecine vétérinaire, Université d'Antananarivo, BP 375, Antananarivo 101, Madagascar

## Abstract

**Background:**

Rabies is a widespread disease in African domestic dogs and a serious public health problem in developing countries. Canine rabies became established in Africa during the 20th century, coinciding with ecologic changes that favored its emergence in canids.

This paper reports the results of a cross-sectional study of dog ecology in the Antananarivo urban community in Madagascar.

A questionnaire survey of 1541 households was conducted in Antananarivo from October 2007 to January 2008. The study addressed both owned and unowned dogs. Various aspects of dog ecology were determined, including size of dog population, relationship between dogs and humans, rabies vaccination.

**Results:**

Dog ownership was common, with 79.6 to 94.1% (mean 88.9%) of households in the six arrondissements owning dogs. The mean owned dog to person ratio was 1 dog per 4.5 persons and differed between arrondissements (administrative districts), with ratios of 1:6.0 in the first arrondissement, 1:3.2 persons in the 2^nd^, 1:4.8 in the 3^rd^, 1:5.2 in the 4^th^, 1:5.6 in the 5^th ^and 1:4.4 in the 6^th ^arrondissement. Overall, there were more male dogs (61.3%) and the male/female sex ratio was estimated to be 1.52; however, mature females were more likely than males to be unowned (OR: 1.93, CI 95%; 1.39<OR<2.69). Most (79.1%) owned dogs were never restricted and roamed freely to forage for food and mix with other dogs. Only a small proportion of dogs (11.7%) were fed with commercial dog food. Only 7.2% of owned dogs had certificates confirming vaccination against rabies. The proportion of vaccinated dogs varied widely between arrondissements (3.3% to 17.5%).

**Conclusion:**

Antananarivo has a higher density of dogs than many other urban areas in Africa. The dog population is unrestricted and inadequately vaccinated against rabies. This analysis of the dog population will enable targeted planning of rabies control efforts.

## Background

In nearly all parts of the world, dogs pose serious human health, socio-economic, political and animal welfare problems. Dogs have been considered to be a nuisance, with their fouling in the streets, dog bites, their persistent barking, particularly at night, and their pack behavior. They may threaten, injure or kill children or adults. Futhermore, given their intimate contact with other animals and man, dogs have the potential to play a significant role as reservoirs and vectors of disease, transferring disease to human and livestock. Dogs are the most important reservoirs of rabies virus in many parts of the world [1,2]. Domestic dogs are by far the most common source of infection to humans [3], with more than 95% of human cases caused by bites from rabid dogs. Rabies has the greatest impact in the developing world, where thousands of people die from rabies annually, and millions receive costly post-exposure vaccination [1,2]. More than 99% of all human deaths from rabies occur in Africa and Asia [4]. Recent studies estimate that canine rabies is responsible for some 55,000 human deaths in these areas each year [4,5].

However, rabies has been neglected in several African countries: the major constraints to effective rabies control are economic and logistic, rather than technical, with poor infrastructure and inadequate resources hampering control programs [6]. Rates of disease transmission depend on the density of the dog population and social behavior that determines the extent of contact. Human rabies can be prevented either by eliminating exposure to rabid animals or by providing exposed persons with prompt local treatment of wounds combined with human rabies immune globulin and vaccine.

In Madagascar, canine rabies is endemic and is an especially serious health problem not only for people living in rural, but also for those living in urban, areas. Every year, rabies strains are isolated by the Rabies National Reference Laboratory (RNRL) in the Institut Pasteur of Madagascar (IPM). Dogs are the main reservoir and vector of the disease. In Antananarivo, the capital city of Madagascar and site of this study, 16 animal samples in 2006 were confirmed positive for rabies by the RNRL, and over 95% of cases of human exposure were due to dog bites (Institut Pasteur of Madagascar, annual report, 2006).

Reports from the rabies post-exposure treatment center in Antananarivo show that about 5,000 patients are exposed (and vaccinated) annually to rabies through mammal bites (Institut Pasteur of Madagascar, annual report, 2006). Most of the wounds were caused by dogs (80% to 90%). Dogs are the principal reservoir of the virus: 499 (70%) of the 714 dogs tested were infected (Institut Pasteur of Madagascar, annual report, 2006). Consequently, rabies is considered to be a major public-health problem in this area.

Rabies epidemiology in the dog reservoir is directly associated to dog ecology; thus, better understanding of dog ecology would be useful for designing appropriate rabies control measures in the dog population [7]. The prevalence and threat of dog rabies in the CUA have not been described, despite easy access to the dog population.

We report a study of dog ecology in Antananarivo (Madagascar) in 2007–2008, in particular estimates of the size and characteristics of the dog population. This information will be valuable for planning more cost-effective rabies vaccination and developing sustainable dog rabies control programs, and to evaluate other threats or public-health problems associated with the presence of dogs.

## Methods

### Study area and interview period

Antananarivo (*Commune Urbaine d'Antananarivo or CUA*) is the capital city of Madagascar. It is located on the central highlands, of which it occupies only a small part. Antananarivo consists of administrative, commercial, industrial and residential areas, and includes patches of agricultural land that are mostly rice fields. The city is divided into six administrative arrondissements, which are divided into *fokontany (n = 192)*, the smallest administrative units. It covers an area of approximately 86.5 km^2^, with a human population estimated in 2007 to be 1,119,235 (Table 1).

**Table 1:** Dog population characteristics, with population size estimated from the dog per person ratios in Antananavario, Madagascar, 2007

	***Total***	**Arrondissements**						**p-value**
		**I**	**II**	**III**	**IV**	**V**	**VI**	
	**n (%)**	**n (%)**	**n(%)**	**n(%)**	**n(%)**	**n(%)**	**n(%)**	
**Households visited**	**1 541**	**296**	**184**	**316**	**266**	**257**	**222**	
*Households without dog*	543	44	34	168	75	107	5	<0.01
	(35.2)	(14.9)	(18.5)	(53.2)	(28.2)	(41.6)	(2.3)	
*Means dogs per household*	1.4	1.3	1.9	0.7	1.3	1.3	1.8	<0.01
*Households with surrounding wall*	511	176	44	78	47	107	59	<0.01
	(35.9)	(59.5)	(30.3)	(33.1)	(17.7)	(41.6)	(26.6)	
**Owned dogs**	**2180**	**388**	**320**	**382**	**360**	**332**	**398**	
*Gender*								
*Male*	1336	217	190	213	239	206	271	
	(61.3)	(55.9)	(59.4)	(55.8)	(66.4)	(62.0)	(68.1)	<0.01
Female	844	171	130	169	121	126	127	
	(38.7)	(44.1)	(40.6)	(44.2)	(33.6)	(38.0)	(31.9)	
*Age*								
Mature	1325	256	220	200	205	214	230	
	(60.8)	(66.0)	(68.8)	(52.4)	(56.9)	(64.5)	(57.8)	<0.01
Young	514	74	79	105	105	69	82	
	(23.6)	(19.1)	(24.7)	(27.5)	(29.2)	(20.8)	(20.6)	
Juvenile	341	58	21	77	50	49	86	
	(15.6)	(14.9)	(6.6)	(20.2)	(13.9)	(14.8)	(21.6)	
*Agressivity*	1126	185	185	267	104	263	122	<0.01
	(51.7)	(47.7)	(57.8)	(69.9)	(28.9)	(79.2)	(30.7)	
*Keep on a leash*	147	43	18	25	17	24	20	<0.01
	(6.7)	(11.1)	(5.6)	(6.5)	(4.7)	(7.2)	(5.0)	
*Guard*	1769	269	269	324	348	236	323	<0.01
	(81.1)	(69.3)	(84.1)	(84.8)	(96.7)	(71.1)	(81.2)	
*Dog scavenge in garbage*	135	3	48	53	0	31	0	<0.01
	(6.2)	(0.8)	(15.0)	(13.9)	-	(9.3)	-	
*History of dog-to-person aggression*	147	36	30	16	19	28	18	<0.01
	(6.7)	(9.3)	(9.4)	(4.2)	(5.3)	(8.4)	(4.5)	
*Vaccinated dogs*	157	24	56	23	13	28	13	<0.01
	(7.2)	(6.2)	(17.5)	(6.0)	(3.6)	(8.4)	(3.3)	
**Unowned dogs**	**274**	**14**	**82**	**35**	**47**	**71**	**25**	
*Gender*								
Male	141	6	49	16	35	25	10	
	(51.5)	(42.9)	(59.8)	(45.7)	(74.5)	(35.2)	(40.0)	<0.01
Female	133	8	33	19	12	46	15	
	(48.5)	(57.1)	(40.2)	(54.3)	(25.5)	(64.8)	(60.0)	
*Age*								
Mature	178	13	54	25	22	48	16	
	(65.0)	(92.9)	(65.9)	(71.4)	(46.8)	(67.6)	(64.0)	0.04
Young	70	1	20	10	19	13	7	
	(25.5)	(7.1)	(24.4)	(28.6)	(40.4)	(18.3)	(28.0)	
Juvenile	26	0	8	0	6	10	2	
	(9.5)	-	(9.8)	-	(12.8)	(14.1)	(8.0)	
*Body condition score*								
Fat	133	4	43	24	13	41	8	
	(48.5)	(28.6)	(52.4)	(68.6)	(27.7)	(57.7)	(32.0)	<0.01
Thin	141	10	39	11	34	30	17	
	(51.5)	(71.4)	(47.6)	(31.4)	(72.3)	(42.3)	(68.0)	
*Social behavior*								
Alone	160	14	46	34	25	29	12	<0.01
	(58.4)	(100.0)	(56.1)	(97.1)	(53.2)	(40.8)	(48.0)	
In group	114	0	36	1	22	42	13	
	(41.6)	-	(43.9)	(2.9)	(46.8)	(59.2)	(52.0)	
*Agressivity*	23	0	8	5	1	8	1	0.23
	(8.4)	-	(9.8)	(14.3)	(2.1)	(11.3)	(4.0)	
*Known in the neighborhood*	250	10	72	30	45	69	24	<0.01
	(91.2)	(71.4)	(87.8)	(85.7)	(95.7)	(97.2)	(96.0)	
**Overall Human Population**	1 119235	226 815	170932	129188	188728	300120	103452	
*Population of the studied households*	10 698	2 337	1 027	1 836	1 877	1 852	1 769	
**Dog Population estimated**								
*Owned dog to person ratio*	1: 4.9	1: 6.0	1: 3.2	1: 4.8	1: 5.2	1: 5.6	1: 4.4	<0.01
*Unowned dog to person ratio*	1:39.0	1:166	1:12.5	1:52.5	1:39.9	1:26.1	1:70.8	<0.01
*Owned dogs estimated*	231 085	37 651	53 330	26 871	36 235	53 721	23 277	
*Unowned Dogs estimated*	29 449	1 318	10 879	2 257	4 167	9 455	1 373	
*Population dog estimated*	260534	38 969	64209	29 128	40 402	63 176	24 650	

Four students at the veterinary department of the faculty of medicine of Antananarivo conducted this study between the 8^th ^of October 2007 and the 12^th ^of January 2008.

### Type of study and collection of data

A cross-sectional study of the population of CUA was carried out using a two-stage cluster sampling technique for each arrondissement: random sampling of the starting *fokontany *in each arrondissement, random selection of the starting point in each *fokontany *and then random selection of direction. The study addressed dogs both associated with and not associated with households. The index household was randomly selected and subsequent households were selected on their proximity to the index household (door to door survey). One member of each household was interviewed. The interview comprised two questionnaires: one to obtain a description of data for owned dogs (dogs with a reference household) and one for the description of the owner's practices with their dogs.

In parallel, unowned dogs (dogs without a reference household, not under human supervision) were studied by asking members of the population about each dog met outside between the index and final household visited. A standard form (Table 1) was used to collect descriptive ecologic data, determining whether the dog was known by spot checks of several inhabitants in the street and examining the dog's characteristics. For each unowned dog, the number of inhabitants interviewed was noted. Age was estimated by each veterinary student using three previously standardized classes: juvenile, young, mature.

If a roaming owned dog was found in the street, its household was identified to collect information about it.

Data collection and questionnaire design were based on the World Health Organization Guidelines for Dog Rabies Control (WHO, 1987). The household information collected included: garbage scavenging by dogs, dog control, dog numbers and gender, other animals kept, and whether any householder had been bitten by an animal in the previous 12 months. Individual dog information included: sex, age, owner, source, function, confinement, food sources, vaccination history, number of litters produced by female dogs and information on the last litter.

The study was continued in each arrondissement until data was obtained for at least 400 dogs (owned or unowned). The sampling technique described above was repeated if an additional fokontany was needed to arrive at the study numbers.

### Statistical analysis

Data gathered were entered into a computer and analyzed using EpiInfo6 software.

The estimated number of owned dogs per district was calculated by multiplying the owned dog per person ratio by the number of inhabitants in the arrondissement.

### Ethical clearance

The study was approved by the Ministry of Health and the National Ethics Committee of Madagascar. Permission and informed consent were obtained from the CUA and study participants.

## Results

### Dog population estimates

The study was conducted across all six arrondissements and included 18 *fokontanys*. The number of households interviewed was 1,541, representing 10,698 persons (9.6% of the population of the selected *fokontanys*). The survey listed 2,180 owned dogs; the overall owned dogs per person ratio was 0.204 (95% CI [0.196–0.211]), or one owned dog for each five inhabitants. Unowned dogs represented 11.1% (274/2454) of the dog sample in CUA, equal to one unowned dog for each 40 inhabitants. However, there were differences between arrondissements: unowned dogs represented 20.4% of the dog population in the second arrondissement but only 3.5% in the first arrondissement (Table 1).

The total dog population in the Urban Municipality of Antananarivo was estimated to be 231,085 owned dogs and 29,449 unowned dogs (Table 1).

### Dog demographic features

#### Age and gender structure

The age structure of the 2,454 dogs for which data were collected in this study is given in Table 2: 61.2% were mature dogs (older than 1 year), 23.8% were young (between 6 months and 1 year old) and 15.0% were juvenile dogs (less than 6 months old). Juvenile dogs were significantly more often owned than mature and young dogs (OR = 1.77, 95% CI: 1.14<OR<2.76 – p < 0.003).

**Table 2:** Age structure of the dog population in CUA in 2007 according to dogs category (Owned or unowned)

**Gender**	**Age group**	**Owned dogs**	**Unowned dogs**	**Total (%)**
Male	*Juvenile*	207 (9.5)	15 (5.5)	222 (9.0)
	*Young*	291 (13.3)	42 (15.3)	333 (13.6)
	*Mature*	838 (38.5)	84 (30.7)	922 (37.6)
Female	*Juvenile*	134 (6.1)	11 (4.0)	145 (5.9)
	*Young*	223 (10.2)	28 (10.2)	251 (10.2)
	*Mature*	487 (22.4)	94 (34.3)	581 (23.7)
**Sex ratio**	*Juvenile*	1.5	1.4	1.5
**(M/F)**	*Young*	1.3	1.5	1.3
	*Mature*	1.7	0.9	1.6

In all categories, there were more male than female dogs; the overall male/female sex ratio was estimated to be 1.5. There was a statistically significant difference (p < 0.002) between owned dogs (male/female sex ratio: 1.59) and unowned dogs (male/female sex ratio: 1.06).

We did not find any difference in gender or category between juvenile and young dogs; however, a higher proportion of unowned dogs than owned dogs were female (OR: 1.93, 95% CI; 1.39<OR<2.69). (Table 2)

The percentage of owned recruited mature females with pregnancy in the last year was 50.9% (248/487). The average number of surviving pups per litter was 3.7 (95% CI: [3.5–4.0]).

#### Unowned dogs in the CUA

The median number of people interviewed for collection of data on unowned dogs was 3 (range 2–10).

Among the 274 unowned dogs observed during this study in CUA, 250 (91.2%) were known by neighborhood residents.

Physical conditions were significantly different between arrondissements (p < 0.01): 48.5% of all unowned dogs in CUA were in good health, with 68.6% in the 3^rd ^arrondissement and only 27.7% in the 4^th ^arrondissement being in good health. No significant differences between gender were found for body condition score. More mature than young or juvenile dogs were in good health (60.1% *vs*. 24.3 and 34.5%, respectively; p < 10^-8^). Unowned dogs known by inhabitants were fatter than dogs that non known (OR: 3.99, 95% CI; 1.35<OR<12.6).

In two arrondissements (first and third), all unowned dogs lived alone, whereas in the other four arrondissements almost half lived in groups.

#### Dog management and handling practices

Among the 2180 owned dogs observed in the six districts of CUA, 20.9% (n = 456) were tied or confined in the house or garden, and 79.1% (n = 1724) lived most of the time outside the property. Household dogs were obtained from various sources, as follows: 36.0% were given by another person, 28.2% were born on the property, 25.7% were bought and 10.1% were found and adopted.

Most (81.1%) owned dogs were considered as guard-dogs, although only 51.7% were aggressive (i.e. barking, growling, with a history of biting).

Only 11.7% of the owned dogs were fed with commercial dog food; 81.2% were fed with family food and 7.1% were not fed by the owners.

Among owned dogs, 25.9% (n = 565) had regular (at least one visit per year) and 32.0% (n = 698) irregular veterinary care, and 42.1% (n = 917) had never been seen by a veterinarian.

During this study, 35.6% (95% CI; [33.5% – 37.6%]) of the owned dogs were reported to have been vaccinated against rabies, but only 21.6% (95% CI; [20.0% – 23.4%]) were regularly vaccinated and 7.2% (95% CI; [6.2% – 8.4%] had a valid certificate or vaccination card.

## Discussion

It has long been recognized that understanding dog population ecology is required for successful rabies control. Ecological studies of dog populations in developing countries are particularly rare although rabies is a major public-health problem in many of these countries. In particular, there has been no reliable investigation of the dog population in Madagascar. This study is the first estimation of dog population size in CUA, the capital city. We obtained a high estimated value for dog population size in this cross-sectional study.

However, our findings were probably subject to information collection bias, particularly concerning unowned dogs, despite having interviewed several inhabitants. For this reason, the age structure of the unowned dogs must be interpreted with care. Age was estimated from both the appearance of the dog and using information obtained from the inhabitants. Futhermore, the mortality rate, principally in the younger group, was unknown and potential increases in the dog population could not be determined.

In Africa, dogs are heavily dependent on humans for food and shelter, so the sizes of dog populations can be correlated with the human population [7]. We found that the dog/human ratio in CUA is high, with one dog per five inhabitants. This result is consistent with what has been reported elsewhere in the world: dog/human ratios of 1:8 in Kenya [8], 1:4.5 in Zimbabwe [9], 1:4.3 in Mexicali [10], and 1:4.6 in Thailand [11]. However, the estimated ratio in CUA is higher than the 1:11 reported for a rural town in South Africa [12], 1:45 for an urban area in Zambia [13], 1:33 for N'Djamena (Chad) [14]. In France, the ratio is one dog per seven inhabitants, and in Asia it is one dog for every eight to 12 persons [2,4].

The proportion of unowned dogs (11% of the total dog population) is the same as that observed in N'Djamena [14]. However, estimates of the unowned dog population may be inaccurate. Most domestic dogs were found to be semi-independent (free-roaming) in terms of their degree of restriction and food sources. Bogel and Joshi estimated, in 1990, that between 30 and 70% of dogs in Africa and Asia were "stray" or "ownerless" [15]. Given that, in Africa, the domestic dogs appears to play a key role in maintenance and transmission of rabies, one important aspect that remains to be studied is the interaction between owned and unowned dogs.

Dogs in CUA are acquired cheaply; indeed, 36% of the surveyed dogs were acquired as gifts and 25.7% were offspring of household bitches. Our findings indicate that the residents of CUA seemed to prefer male dogs rather than females, consistent with reports that male dogs predominate in other parts of the world [9,16-18]. This preference appears to be due to the belief that male dogs make better guards and hunters. The needs for guarding property and protecting livestock in Africa in relation to the increasing urban violence could explain the high human:dog ratio found in this study. Guard duties have been identified as the primary reason for keeping dogs in a number of other countries including Zimbabwe [9], Zambia [13], Mexico City [19], Philippines [20], and the city of Guayaquil in Ecuador [18].

We found that the females were more common in the mature unowned dog population, suggesting that the unowned dog population can grow rapidly. As a consequence, controlling the unowned dog population in CUA may be difficult. This problem has been observed in African countries where dog populations are characterized by high turn-over rates and where rabies has been attributed to rapidly growing dog populations (typically 5–10% per annum [4]).

Human behavior facilitates the survival of unowned dogs, and, in reality, "stray dogs" belong to the community; indeed, unowned dogs known by residents are in good health and were the most mature.

Most unowned dogs in the CUA lived in groups, facilitating the control of rabies in these animals by oral mass vaccination. Mass vaccination is the preferred approach by WHO experts, given that dog elimination programs may, perversely, be counter-productive and reduce the proportion of immunized individuals in a population [2].

The rabies immunization coverage of owned dogs in CUA is less than 40% (6% to 37%) and is therefore not sufficient to prevent transmission. According to some authors, vaccination coverage of 70% is considered to be necessary to prevent outbreaks of dog rabies [2]. In addition, many (79%) owned dogs are left to roam freely and, as a result, are at risk of contracting rabies virus and other types of zoonosis. This is a dangerous public health situation, increasing the infectious disease threat. So, mass vaccination campaigns should be advocated and dog owners need to be provided with material and financial assistance care for their dogs, especially to immunize them against rabies. Since nearly 90% of the dog population is owned and can be reached by their owner, a mass vaccination campaign using a parenteral vaccine may be the best approach as it is one of the least expensive. Models of the transmission of canine rabies indicate that rabies can be eradicated if 70% of a dog population is repeatedly vaccinated [21].

## Conclusion

Rabies is a persistent problem in Madagascar. This study shows that the dog population in CUA is large and poorly supervised, highly dynamic, and inadequately vaccinated against rabies. Consequently, there is a real risk of rabies spreading through the human population.

The WHO advocates vaccination of owned dogs to cut rabies transmission. In CUA, any program of rabies elimination must involve dog vaccination if it is to be effective. In addition, the population of CUA needs to be properly informed about the positive effects of systematic and regular dog vaccination and the importance of dog ownership and restricting their movements. Attitudes need to be changed to ensure sufficient vaccination coverage. Indeed, the best way to prevent rabies from being contracted by a pet owner is to keep domesticated animals vaccinated.

If rabies is not eliminated in developing countries, the financial costs of prevention of human disease will increase significantly.

## Authors' contributions

MR participated in the planning of the study and data analysis, and JHR participated in the planning of the study. SR, HR, MPA and FAR were the principal investigators, participated in the planning and execution of the study, and performed data entry and data analysis. VR was the project coordinator and participated in the planning, data analysis and writing. All authors read and approved the final manuscript.
